# Antioxidant Capacities, Phenolic Profile and Cytotoxic Effects of Saxicolous Lichens from Trans-Himalayan Cold Desert of Ladakh

**DOI:** 10.1371/journal.pone.0098696

**Published:** 2014-06-17

**Authors:** Jatinder Kumar, Priyanka Dhar, Amol B. Tayade, Damodar Gupta, Om P. Chaurasia, Dalip K. Upreti, Rajesh Arora, Ravi B. Srivastava

**Affiliations:** 1 Defence Institute of High Altitude Research, Defence Research & Development Organisation, Leh-Ladakh, Jammu & Kashmir, India; 2 Medicinal and Aromatic Plants Laboratory, Radiation Biotechnology Group, Institute of Nuclear Medicine and Allied Sciences, Defence Research and Development Organisation, Delhi, India; 3 Office of the Director General-Life Sciences, DRDO Bhawan, New Delhi, India; 4 Lichenology Laboratory, Plant Biodiversity and Conservation Biology Division, CSIR- National Botanical Research Institute, Lucknow, Uttar Pradesh, India; University of Sassari, Italy

## Abstract

Fourteen saxicolous lichens from trans-Himalayan Ladakh region were identified by morpho-anatomical and chemical characteristics. The n-hexane, methanol and water extracts of the lichens were evaluated for their antioxidant capacities. The lichen extracts showing high antioxidant capacities and rich phenolic content were further investigated to determine their cytotoxic activity on human HepG2 and RKO carcinoma cell lines. The ferric reducing antioxidant power (FRAP), 2,2′-azinobis-(3-ethylbenzothiazoline-6-sulfonic acid) diammonium salt (ABTS), 1,1-diphenyl-2-picrylhydrazyl (DPPH) and nitric oxide (NO) radical scavenging capacities and β-carotene-linoleic acid bleaching property exhibited analogous results where the lichen extracts showed high antioxidant action. The lichen extracts were also found to possess good amount of total proanthocyanidin, flavonoid and polyphenol. The methanolic extract of *Lobothallia alphoplaca* exhibited highest FRAP value. Methanolic extract of *Xanthoparmelia stenophylla* showed the highest ABTS radical scavenging capacity. The n-hexane extract of *Rhizoplaca chrysoleuca* exhibited highest DPPH radical scavenging capacity. Highest antioxidant capacity in terms of β-carotene linoleic acid bleaching property was observed in the water extract of *Xanthoria elegans*. Similarly, *Melanelia disjuncta* water extract showed highest NO scavenging capacity. Among n-hexane, methanol and water extracts of all lichens, the methanolic extract of *Xanthoparmelia mexicana* showed highest total proanthocyanidin, flavonoid and polyphenol content. From cytotoxic assay, it was observed that the methanolic extracts of *L. alphoplaca* and *M. disjuncta* were exhibiting high cytotoxic effects against cancer cell growth. Similarly, the water extract of *Dermatocarpon vellereum*, *Umbilicaria vellea*, *X. elegans* and *M. disjuncta* and the methanolic extract of *M. disjuncta* and *X. stenophylla* were found to possess high antioxidant capacities and were non-toxic and may be used as natural antioxidants for stress related problems. Our studies go on to prove that the unique trans-Himalayan lichens are a hitherto untapped bioresource with immense potential for discovery of new chemical entities, and this biodiversity needs to be tapped sustainably.

## Introduction

Free radicals having one or more unpaired electrons are generated as a by-product in normal or pathological cell metabolism. Reactive oxygen species (ROS) react swiftly with free radicals to become radicals themselves thereby starting free radical chain reaction. Superoxide anion (O^2–^), hydrogen peroxide (H_2_O_2_), hydroxyl radical (HO^•^) and singlet oxygen (^1^O_2_) are the various forms of reactive oxygen species [1], [2]. Excess ROS in our body damages biological molecules leading to the development of degenerative diseases such as premature aging, heart diseases, cancer, inflammation, diabetes, genotoxicity, arthritis and many more [3], [4]. Exogenous sources of free radicals *viz.* tobacco smoke, ionizing radiation, certain pollutants, organic solvents, pesticides etc. trigger the process of generation of ROS within the body [5], [6]. The most efficient way to exterminate free radicals that cause the oxidative stress is through antioxidant supplementation.

Antioxidants are compounds which can impede the oxidation process by reacting with free radicals, chelating catalytic metals and scavenging oxygen in biological systems [7]. Hence, antioxidants are of prime importance in preventing various pathophysiological dysfunctions and diseases [8]–[10]. However, the synthetic antioxidants *viz.* butylated hydroxyanisole (BHA), butylated hydroxytoluene (BHT), tertbutylhydroquinone (TBHQ) and propyl gallate (PG) have been reported to exert toxic effects [11]. Consequently, there is a growing interest towards finding natural antioxidants of plant resources without any undesirable effect [1], [6]. Ethnopharmacological and *in vitro* studies on medicinal plants and vegetables strongly supports the fact that phytoconstituents with antioxidant capacity are capable of exerting protective effects against oxidative stress in biological systems [12], [13]. Therefore, it is of prime importance to utilize natural antioxidants for their protective effect against oxidative stress and physiological dysfunctions [14], [15]. Natural antioxidants obtained from various resources such as plants, micro- and macro-algae, macromycetes and lichens have become one of the major research areas in recent times. In the quest for novel natural antioxidant sources, our prime interest has focused on lichens.

Lichens, the unanimously disseminated organisms occurring in the most adverse and varied geo-climatic circumstances ranging from the ice-capped poles to the tropics and from the plains to the highest mountains on earth and substrates, encompass the most inimitable group of organisms in nature, growing simultaneously in a close successful symbiotic alliance of two distinct organisms, an exhabitant fungus (the mycobiont) and the photoautotrophic partner algae (the phycobiont) [16], [17]. Lichenology remains rather neglected throughout the world, though collectively with mosses they form the omnipresent organisms in ecosystem casing over 10% of the earths’ terrestrial habitats predominantly at higher elevations [18], [19]. Lichens are known to have therapeutic effects on various diseases in traditional system of medicine of many countries. A number of factors such as specific and extreme habitat, slow growth and long life are the basis for the production of diverse bioactive compounds having protective functions against several physical and biological influences [20], [21]. Various scientific reports suggest that the lichens have antimicrobial, antiviral, antitumor, antiinflammatory, analgesic and antipyretic, antiproliferative and antiprotozoal potentials [22], [23]. The antioxidant properties of lichens and their secondary metabolites are poorly known and in recent time the potential of lichens as resources of natural antioxidants have been investigated by researchers [24]–[29] and only few of them have been reported to have promising antioxidative potential [25], [30]. In the world, India is a rich centre of lichens diversity, contributing nearly 15% of the 13500 species of lichens so far recorded [31]. Therefore, it is necessary to explore the remaining unreported lichen species of this country.

The Indian Himalayan region is well known for its varied characteristic ecosystems endowed with rich floristic and faunal wealth. Plants of the Himalayan region are widely used in traditional medical systems both as prophylactics and therapeutics for high altitude maladies and a number of natural products and botanical supplements have been developed from our institutes, which are useful to combat these problems. These products have been reported to possess high nutritional and antioxidant properties. Extensive research work was carried out by the previous investigators to explore the medicinal and aromatic plants of trans-Himalaya [32]–[34]. However, research reports on lichen flora of this megadiversity hotspot of the world are very limited. In our recent study, we have reported the diversity of lichens along altitudinal and land use gradients from trans-Himalyan region. The harsh climatic condition of trans-Himalayan Ladakh, along with its fragile ecosystem, desert soil and rocky habitat constitute ecological niche for lichen species [35], [36]. In the present investigation, we first aimed to study the antioxidant capacity and phenolic profile of lichens from this region. Out of all lichen families under investigation, lichen species of families *viz.* Umbilicariaceae (*Umbilicaria esculenta*, *U. vellea*, *U. muhlenbergii* and other *Umbilicaria* spp.), Teloschistaceae (*Xanthoria parietina*, *X. elegans*) and Parmeliaceae (*Xanthoparmelia chlorochroa*, *X. conspersa*, *Parmelia tinctorum*, *P. nilgherrensis*, *P. reticulata* and *P. sancti-algelia*) are used as food ingredients and for medicinal purposes in different parts of the world [37]–[43].

However, the medicinal and therapeutic effects with respect to antioxidant capacities, cytotoxic activity and phenolic composition of different extracts of saxicolous lichens *viz. Dermatocarpon vellereum, Umbilicaria vellea, Rhizoplaca chrysoleuca, Rhizoplaca melanophthalma, Pleopsidium flavum, Xanthoparmelia mexicana, Acarospora badiofusca, Xanthoria elegans, Lecanora frustulosa, Lobothallia alphoplaca, Physconia muscigena, Melanelia disjuncta, Xanthoparmelia stenophylla and Peccania coralloides* from trans-Himalayan cold desert of Ladakh have not been reported till date. For this rationale, the present investigation was desinged to estimate the *in vitro* antioxidant capacities and phenolic profile of n-hexane, methanol and water extracts of the aforementioned fourteen saxicolous lichens from trans-Himalaya. The lichen extracts showing high antioxidant capacities and rich phenolic content were further investigated to determine their cytotoxic activity on human hepatocellular carcinoma HepG2 and colon carcinoma RKO cells.

## Materials and Methods

### Chemicals and reagents

1,1-Diphenyl-2-picrylhydrazyl (DPPH), 2,2′-azinobis-(3-ethylbenzothiazoline-6-sulfonic acid) diammonium salt (ABTS), 2,4,6-tripyridyl-*s*-triazine (TPTZ), ferrous sulfate (FeSO_4_.7H_2_O), aluminium chloride (AlCl_3_), sodium acetate (C_2_H_3_NaO_2_), sodium carbonate (Na_2_CO_3_), potassium persulfate (K_2_S_2_O_8_), potassium chloride (KCl), ferric chloride (FeCl_3_), sodium nitroprusside {Na_2_[Fe(CN)_5_NO].2H_2_O}, sulfanilic acid (C_6_H_7_NO_3_S), butylated hydroxytoluene (BHT), ascorbic acid, gallic acid, quercetin and catechin were purchased from Sigma-Aldrich (St. Louis, MO, USA). RPMI medium 1640, penicillin, streptomycin, trypsin-EDTA, sulphorhodamine B, trichloroacetic acid, acetic acid, Tris base and all other chemicals used for the cytotoxicity assay were of analytical grade and also purchased from Sigma-Aldrich (St. Louis, MO, USA). Folin Ciocalteu's phenol reagent, vanillin, silica gel precoated aluminium TLC plates (20×20 cm), hydrochloric acid, sulphuric acid, methanol, n-hexane, chloroform, ethanol, toluene, dioxane, acetic acid, diethyl ether, formic acid and sodium carbonate were purchased from Merck Chemical Supplies (Merck KGaA, Darmstadt, Germany). All the other chemicals used including solvents were of analytical grade.

### Ethics statement

All necessary permits were obtained for the described field studies and lichen collection. The permit was issued by Dr. B. Balaji (IFS), Divisional Forest Officer, Leh Forest Division, Jammu & Kashmir, India.

### Sample collection

Amongst the fourteen lichen specimens thirteen lichens *viz. Dermatocarpon vellereum* (Zschacke), *Umbilicaria vellea* (L.) Ach. *em.* Frey, *Rhizoplaca chrysoleuca* (Sm.) Zopf, *Rhizoplaca melanophthalma* (DC) Leuck. & Poelt, *Pleopsidium flavum* (Bell.) Korb., *Xanthoparmelia mexicana* (Gyeln.) Hale, *Acarospora badiofusca* (Nyl.) Th. Fr., *Xanthoria elegans* (Links.) Th. Fr., *Lecanora frustulosa* (Dick.) Ach., *Lobothallia alphoplaca* (Wahlenb. ex Ach.), *Physconia muscigena* (Ach.) Poelt., *Melanelia disjuncta* (Essl.), *Xanthoparmelia stenophylla* (Ach.) Ahti & Hawksw. and *Peccania coralloides* (Massal.) Massal. were collected from the northward slope of hill [native primary scrubland located 34°08′08.86"N, 77° 34′36.0"E and 3530 m altitude above sea level (ASL)], Indus valley, Leh-Ladakh, J&K, India, in the peak winter period (minimum temperature −28°C, maximum temperature −3°C) of January, 2012. *Umbilicaria vellea* was collected from the location near Chang-La Top (barren cold desert located 34°02′36.05"N, 77°56′26.6"E and 5189 m altitude ASL), Changthang valley, Leh-Ladakh, J&K, India in May, 2012. Lichens were carefully collected using scalpel and forceps following standard procedure. Then dust, soil, and rock debris were removed by using brushes, forceps and needles, shade dried to a constant weight (dry weight, dw) and lichen samples were kept at room temperature until extraction in the sterile petriplate (Tarsons 10.0 cm TPX, 461030, Tarsons Products Pvt. Ltd., Kolkata, India).

### Identification, morpho-anatomical and colorimetric characterisation of lichen species

Lichens were identified morpho-anatomically using a stereomicroscope, light microscope, and chemically with the help of color reactions, UV light and standardized thin-layer chromatography (TLC) [44], [45]. The precise identification of lichen species on the basis of occurrence of chemical compounds was performed by spot test and thin layer chromatography (TLC). Silica gel was used as stationary phase while different solvent systems were used as mobile phase. Three solvent systems *viz.* solvent system A (toluene: dioxane: acetic acid:: 180: 60: 8), solvent system B (hexane: diethyl ether: formic acid:: 130: 100: 20) and solvent system C (toluene: acetic acid:: 200: 30) were used as mobile phase. TLC was performed in accordance with the previous reports for lichen identification [44], [45]. Identification was done using relevant key and monographs [46], [47]. The voucher specimens are preserved in the Lichen Herbarium (LWG), CSIR–National Botanical Research Institute (NBRI), Lucknow, India.

### Sample preparation and extraction

Lichen specimens were air dried at room temperature and ground separately to powder. Finely ground dry thalli of all lichen species (170.4 mg to 330 mg depending upon their abundance) were sequentially extracted in 1 ml of solvents *viz.* n-hexane, methanol and water by vortexing for 10 min, and then centrifugation at 10,000 rpm for 15 min. This cycle of extraction for each lichen sample was at 10°C and repeated two times as each solvent added freshly to ensure complete extraction [48]. The extracts were filtered and transferred in new micro centrifuge tubes until debris was completely removed. The extracts was then concentrated by keeping in water bath at 20–40°C. These concentrated extracts were kept at −20°C for further use.

### Antioxidant capacities

#### Ferric reducing antioxidant power (FRAP) assay

Ferric reducing antioxidant power (FRAP) assay was performed as suggested by previous investigators [49]. A total of 75 µl of extract and 225 µl of distilled water were added to 2.25 ml of freshly prepared FRAP reagent [10 parts of 300 mM sodium acetate buffer at pH 3.6, 1 part of 10 mM 2,4,6-tri (2-pyridyl)-*s*-triazine (TPTZ) solution and 1 part of 20 mM FeCl_3_.6H_2_O]. The content was incubated in the dark for 30 min. The increase in absorbance with the formation of colored product (ferrous tripyridyltriazine complex) was measured at 593 nm. The antioxidant capacity of the lichen extract was determined based on a calibration curve plotted using FeSO_4_.7H_2_O at a concentration ranging between 0.125 and 2 mM. The calibration equation for FeSO_4_.7H_2_O was y = 0.464×−0.0114, R^2^ = 0.9996, where *x* is the concentration of FeSO_4_.7H_2_O mM/l and *y* is the absorbance at 593 nm. Results were expressed in mM Fe (II)/g of extract.

#### ABTS radical scavenging activity

The ABTS assay was performed according to the previously established protocol [50]. The stock solution was prepared by mixing equal volumes of 7 mM ABTS solution and 2.45 mM potassium persulfate solution followed by incubation for 12 h at room temperature in the dark to yield a dark-colored solution containing ABTS^•+^ radicals. Working solution was prepared freshly before each assay by diluting the stock solution by mixing of stock solution to 50% methanol for an initial absorbance of about 0.700±0.02 at 745 nm, with temperature control set at 30°C. Free radical scavenging activity was assessed by mixing 30 µl of different fractions (different concentration in respective solvents) with 300 µl of ABTS working standard. The decrease in absorbance was measured after 6 min at 30°C. Quercetin and ascorbic acid were used as positive control. The scavenging activity was estimated based on the percentage of ABTS radicals scavenged by the following formula:
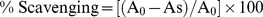



Where, A_0_ was absorption of control, A_S_ was absorption of tested extracts and standards.

The half maximal inhibitory concentration (IC_50_) for scavengers (radical scavenging concentration_50_ or RSa_50_) was calculated [34], [51]. The RSa_50_ value was determined by plotting the scavenging capacity against the logarithm of sample concentration.

#### Inhibitory effect on 1,1-diphenyl-2-picrylhydrazyl radical (DPPH)

We followed the method suggested by previous investigators [52], [53] for estimating the DPPH radical scavenging capacity of lichen extracts. The 0.1 mM solution of DPPH in methanol was prepared and 500 µl of the solution was reacted with 25 µl of the extracted lichen sample. A control was reacted with 25 µl of solvent instead of the extract. The mixture was incubated at room temperature for 30 min before the shrink in absorbance at 517 nm was recorded. Quercetin and ascorbic acid were used as positive control. The percentage inhibition was calculated as follows:
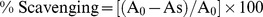



Where, A_0_ was absorption of control, A_S_ was absorption of tested extracts and standards.

The IC_50_ values were also calculated as described in the previous section.

#### β-carotene-linoleic acid bleaching assay

For the β-carotene-linoleic acid bleaching assay, the previously depicted method [54] was used. A stock solution of β-carotene and linoleic acid was prepared as follows: 0.5 mg of β-carotene was dissolved in 1 ml of chloroform, 25 µl of linoleic acid and 200 mg of Tween 20 were added. After chloroform evaporation under vacuum, 100 ml of distilled water was added to the residue. An aliquot of 500 µl of each extract or BHT (in different concentration) was pipetted into separated test tubes and 5 ml of the previous mixture was added. The test tubes were incubated for 2 h (120 min) at 50°C together with the control sample. The absorbance was measured at 470 nm at the beginning (t = 0 min) and after the experiment (t = 120 min). BHT was used as positive control. The antioxidant capacity was calculated as percentage inhibition of oxidation using the following equation:
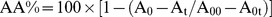
where AA% is percent of antioxidant activity, A_0_ is the absorbance at beginning of reaction, A_t_ is the absorbance after 2 h with sample extract, A_00_ is the absorbance at beginning of reaction without sample extract and A_0t_ is the absorbance after 2 h without sample extract. The IC_50_ or RSa_50_ values were also calculated as described in the previous section.

#### Nitric oxide (NO) scavenging capacity

The method of Wang et al. (2009) [55] was used to assay the scavenging activity of extracts on nitric oxide. The reaction solution (100 µl) containing 10 mM sodium nitroprusside in PBS (pH 7.0) was reacted with 10 µl extracts of different concentrations. The reaction mixture was then incubated at 37°C for 1 h. Thereafter 50 µl aliquot was mixed with 50 µl of Griess reagent [1.0 ml of sulfanilic acid reagent {0.33% prepared in 20% glacial acetic acid at room temperature for 5 min with 1 ml of naphthyethylenediamine dihydrochloride (0.1%, w/v)}] and the absorbance at 540 nm was measured. Percent inhibition of nitric oxide produced was calculated by comparing with the absorbance value of the negative control (10 mM sodium nitroprusside and PBS). BHT was used as positive control. The IC_50_ or RSa_50_ values were also calculated as described in the previous section.

### Phytochemical compositions

#### Determination of total proanthocyanidin content (TPAC)

Determination of proanthocyanidin was performed according to the method of Sun et al. (1998) [56]. A volume of 0.5 ml extract solution was allowed to react with 3 ml of 4% vanillin-methanol solution and 1.5 ml hydrochloric acid. Then the mixture was allowed to stand for 15 min. The absorbance was measured at 500 nm at 30°C. Total proanthocyanidin content was calculated as catechin equivalent (CAE) in mg/g of extract using the following equation based on the calibration curve: y = 0.0015×−0.0209, R^2^ = 0.9975, where x was the absorbance and y was the CAE. Extract samples were evaluated at a final concentration of 100 µg/ml.

#### Determination of total flavonoid content (TFC)

Total flavonoid was analyzed with the method of Ordonz et al. (2006) [57]. Briefly, 100 µl of sample and 2% AlCl_3_ in ethanol solution were allowed to react. The reaction mixture was incubated for 1 h at room temperature and the absorbance was measured at 420 nm. Total flavonoid content was calculated as quercetin equivalent (QAE) in mg/g of extract by using the following equation based on the calibration curve: y = 0.0235x−0.1803, R^2^ = 0.9994, where *x* was the absorbance and *y* was the QAE at a final concentration of 100 µg/ml.

#### Determination of total polyphenol content (TPC)

Total polyphenol content (TPC) in extract was determined by Folin Ciocalteu's colorimetric method as described by Gao et al. (2000) [58]. Extract solution (10 µl) was mixed with 20 µl of 10% Folin Ciocalteu's reagent and 200 µl of H_2_O, and incubated at room temperature for 3 min. After that 100 µl of sodium carbonate (20%, w/v) was added to the reaction mixture. The content was incubated at room temperature in the dark for 30 min and absorbance was measured at 765 nm. Estimation of total polyphenol content was calculated as gallic acid equivalent (GAE) in mg/g of extract on the basis of calibration curve of gallic acid, y = 0.0104× −0.0589, R^2^ = 0.9978, where x was absorbance and y was GAE at a final concentration of 100 µg/ml.

### Cytotoxic effects of lichen extracts

#### Cell cultures

Human hepatocellular carcinoma (HepG2) and colon carcinoma (RKO) cell lines acquired in liquid nitrogen (−180°C) were procured from American Type Culture Collection (ATCC), USA. Cells were maintained in RPMI medium 1640 (supplemented with 10% heat-inactivated fetal bovine serum, 100 units/ml of penicillin and 100 µg/ml of streptomycin, pH 7.4) at 37°C in 5% CO_2_ and 95–100% humidified air atmosphere.

#### Morphological determination of cell toxicity

Logarithmically growing cells were seeded in 96 flat-well plate, (HepG2 cells 3,000/well and RKO cells 3,000/well) and incubated for 24 h to have a partial monolayer. The extracts of lichens were dissolved in RPMI medium 1640 and cells were treated with different concentration (29.26, 43.90, 65.84, 98.77, 148.15, 222.22 and 333.33 µg/ml) of extracts. Thereafter, cells were incubated at 37°C in a 5% CO_2_ and 95–100% humidified air atmosphere (Touch 190S, LEEC, Nottingham, UK). Microscopic examination was performed (10X eyepiece and 10X objective lenses) using inverted phase contrast microscope (Dewinter Optical, Inc., Delhi, India). The changes in cellular morphology following different treatments were acquired at different time intervals (24, 48 and 72 h).

#### Sulphorhodamine B (SRB) assay for cytotoxicity screening

The Sulphorhodamine B (SRB assay) was performed for evaluating cellular growth and colorimetric determination of cytotoxicity of lichen extracts was performed [59], [60]. The cells were harvested with 0.5% trypsin-EDTA. At zero time after extract inoculation in the cell culture and after 72 h of incubation at 37°C in 5% CO_2_, in humidified incubator (95–100% humidity), the SRB assay was performed. At both points of time, the medium was removed and the cells were fixed with trichloroacetic acid (20%, w/v) at 4°C for 1 h, stained for 30 min with SRB (0.4%, w/v) dissolved in 1% acetic acid for 30 min and washed four times with 1% acetic acid. The protein-bound dye was made solubilised with 10 mmol/L tris base, pH 10.5 and the absorbance was recorded at 565 nm using spectrophotometer (Spectramax M2^e^, Molecular Devices, Germany).

The percentage growth (PG) at each extract concentration level was calculated as:
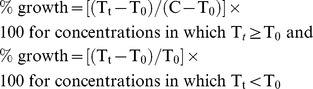



The 50% growth inhibition concentration (GI_50_, µg/ml) was the concentration where {(T_t_−T_0_)/(C−T_0_)} ×100 = 50. Total growth inhibition concentration (TGI, µg/ml) was the concentration where {(T_t_ −T_0_)/(C−T_0_)} ×100 = 0 = PG. Lethal concentration that kills 50% of the cells (LC_50_, µg/ml) was the concentration level where {(T_t_−T_0_)/T_0_} ×100 = −50. These parameters were determined following established reports [61]–[63] and expressed by plotting graph of cell growth against different concentration of each lichen extract. Where, T_0_ =  absorbance of the extract treated cells just after time zero, and T_t_ =  absorbance of the extract treated cells after 72 h, C =  absorbance of control (cells untreated with extract) after 72 h.

### Statistical Analysis

All the experimental results were expressed as mean ± standard deviation (SD) using statistical analysis with SPSS 17.0 (Statistical Program for Social Sciences, SPSS Corporation, Chicago, IL) version. Analysis of variance (ANOVA) in a completely randomised design, Duncan's multiple range test and Pearson's correlation coefficients were performed to compare the data. Post hoc analysis was performed using Neuman Keuls Test, and values with *p*<0.05 were considered significant.

## Results

### Morpho-anatomical features of identified lichen species

**Figure 1:**
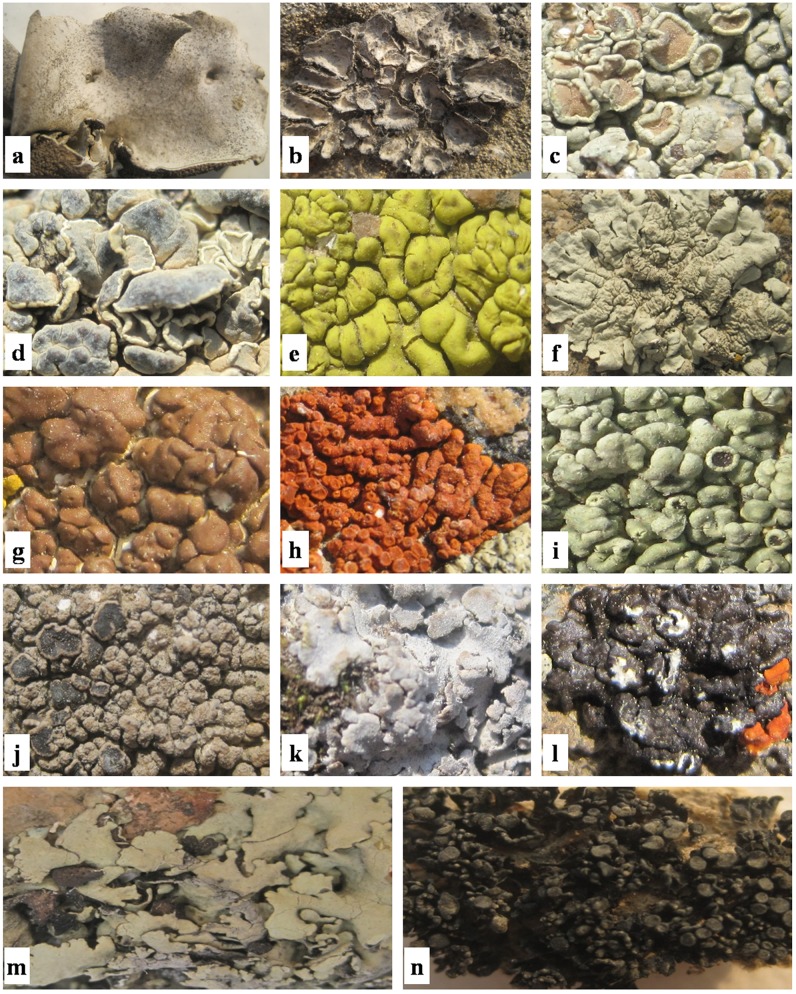
Thallus of lichen species studied in the present investigation. **a**: *Dermatocarpon vellereum*; **b**: *Umbilicaria vellea*; **c**: *Rhizoplaca chrysoleuca*; **d**: *Rhizoplaca melanophthalma*; **e**: *Pleopsidium flavum*; **f**: *Xanthoparmelia mexicana*; **g**: *Acarospora badiofusca*; **h**: *Xanthoria elegans*; **i**: *Lecanora frustulosa*; **j**: *Lobothallia alphoplaca*; **k**: *Physconia muscigena*; **l**: *Melanelia disjuncta*; **m**: *Xanthoparmelia stenophylla*; **n**: *Peccania coralloides*.

In the present study lichen specimens were collected from Chang-La Top of Changthang valley and northward slope of hill of Indus valley, Ladakh, India. The collected lichens specimens were identified using standardized analytical techniques, significant lichen identification keys and monographs [44]–[47]. The thallus structure of all lichen species has been illustrated in Fig. 1. The data on morphological, anatomical, colorimetric and taxonomic charateristics recorded during the identification for fourteen lichen species have been depicted in Table 1 and Table 2. Wide variations in the parameters were observed among the investigated lichen species. Three lichen species additions *viz. Lobothallia alphoplaca*, *Rhizoplaca melanophthalma* and *Xanthoparmelia mexicana* were reported to the area near the northward slope of hill of Indus valley for the first time as per our previously reported work [35].

**Table 1:** Taxonomic description of lichen species.

*Dermatocarpon vellereum* Zschacke (Family: Verrucariaceae)
Thallus saxicolous, foliose, umbilicate, monophyllous, leathery, upper side light brownish to brownish red, white to dark pruinose, lower side black, with dense, thick, stumpy, coralloid rhizinomorphs. Perithecia pale red; ascospores ellipsoid, 9-12×(5) 6–9 µm.
*Umbilicaria vellea* (L.) Ach. em. Frey (Family: Umbilicariaceae)
Thallus saxicolous, monophyllous, umbilicate, grey to blackish grey, smooth. Areolate, pruinose, lower side black, rhizomorphs dimorphic. Apothecia black, gyrodiscus, ascospores simple, colourless 8.5–13×6.8–10 µm.
*Rhizoplaca chrysoleuca* (Sm.) Zopf (Family: Lecanoraceae)
Thallus saxicolous, foliose, umbilicate, monophyllous or polyphyllous, monophyllous upto 3 cm across, polyphyllous with thick lobes united by a stalk at centre; lower side brown at centre, bluish black in outer part. Apothecia to 5 mm in diameter, disc orange- red to red, pruinose; ascospores 8.5–12×3.5–6 µm. Medulla K−, Pd+ yellowish. Placodialic acid was present.
*Rhizoplaca melanophthalma* (DC) Leuck. & Poelt (Family: Lecanoraceae)
Thallus saxicolous, peltate, monophyllous, up to 3 cm across, lobes round to crenate, lower side reddish brown. Apothecia to 3 mm in diameter, sessile, disc bluish brown to black, spores 9–11.5×5–5.5. Medulla P+ yellow, Placodialic and rarely psoromic acid were present.
*Pleopsidium flavum* (Bell.) Korb. (Family: Acarosporaceae)
Thallus saxicolous, marginally lobate, yellow, effigurate, ± areolate, marginal lobes of thallus rough, subconvex, scabrid and 1.5–2 mm long. Apothecia plane, solitary, to 0.1–1 mm in diameter, immersed in areolae, disc plane brown, margin thick, persistent, spores simple, hyaline, ellipsoid, 4–5×1.7–2 µm.
*Xanthoparmelia mexicana* (Gyeln.) Hale (Family: Parmeliaceae)
Thallus saxicolous, foliose; lobes 1.5–3 mm wide, black rimmed; upper side yellow- green, isidiate; isidia subglobose to cylindrical, simple to coralloid branched, black tipped; lower side brownish, rhizinate; medulla white.
*Acarospora badiofusca* (Nyl.) Th. Fr. (Family: Acarosporaceae)
Thallus wide spreading, areolate, areoles rather small, 0.5–1.5 mm wide, ± rounded, numerous, at times wavy, irregular, rarely imbricate, flat or ± convex, reddish brown, algal layer continuous. Apothecia 0.4–2 mm diameter, mostly single, rarely 2 to 4 contiguous per areoles, rounded or gyrose-contorted, sessile, thalline exiple usually distinct, ± elevated, entire, concolorous with disc, hymenium 60–75(−90) µm tall, disc flat-convex, oftened roughened, usually red brown to brown black, always darker than thallus, paraphyses 2.5–3 µm wide at base, 4–5 µm at tips, asci 200 spored, spores 3–6×1.5–2.5 µm, ellipsoid.
*Xanthoria elegans* (Links.) Th. Fr. (Family: Teloschistaceae)
Thallus saxicolous, foliose, suborbicular, lobes radiating, compact, convex, nodulose with densely crowded apothecia in central part, upper side orange-red to reddish brown, lower side grey, medulla white, ± hollow. Apothecia up to 1 mm in diammeter, spores 12–16 (−18)×6–8 (−10) µm with 4–5 µm thick transverse septum. Upper surface K+ purple, Pd−, C−, I−. Parietin, fallacinal, emodin, teloschistin, parietinic acid found to be present. Chemosyndrome A was also detected.
*Lecanora frustulosa* (Dick.) Ach. (Family: Lecanoracea)
Thallus placodioid, closely adpressed, greenish yellow to yellow brown, margin pale or whitish above, 0.3–0.7 mm wide,1.0–3.8 mm long lower surface pale brown to black. Apothecia sessile, densely aggregate in centre of the thallus, 0.4–1.8 mm diameter, spores 8 per ascus, ellipsoidal, 9.0–16.0×4.5–7.0 um. Thallus and apothecial margin K−, C−, KC+ yellowish, PD−. Usnic acid and zeorin was reported in the species.
*Lobothallia alphoplaca* (Wahlenb. ex Ach.) Hafellner (Family: Megasporaceae)
Thallus saxicolous, crustose, whitish- grey, verrucose areolate centrally, radiating laciniae marginally, hollow. Apothecia rounded, 0.5–2.0 (−2.5) mm diameter, disc plane to slightly convex, dark brown to brown black, margin whitish, smooth, ascospores 8 per ascus, 8–12×4–8 µm. Thallus K+ yellow then red, Pd+ yellow-orange, C−, KC−. Norstictic and salazininc acids were detected.
*Physconia muscigena* (Ach.) Poelt. (Family: Physciaceae)
Thallus tericolous or muscicolous, to 10 cm across, lobes 1–1.5 (apically 3) mm wide; upper side brownish, lacking isidia or soredia; lower side black; rihizines squarrosely branched. Apothecia to 5 mm in diameter, ascospores 23–32 (−35)×12–16 µm.
*Melanelia disjuncta* Essl. (Family: Parmeliacea)
Thallus appressed, lobes flat to slightly convex or concave, contiguous, upper surface usually dark olive brown, dark brown or blackish, pseudocyphellae small, submarginal, sorediate, lower surface, moderately rhizinate. Apothecia infrequent, sessile, margin sorediate, rugose, pseudocyphellate, spores ellipsoid, 9–12.5×5–7 µm. Perlatolic and stenosporic acid were present.
*Xanthoparmelia stenophylla* (Ach.) Ahti & Hawksw. (Family: Parmeliaceae)
Thallus saxicolous, foliose, pulvinate, lobes sublinear, 1.2–5 mm wide, brownish at apices, secondary lobules developing at centre, often erhizinate, upper side yellow-green, lacking isidia and soredia, lower side brownish, rhizinate.
*Peccania coralloides* (Massal.) Massal. (Family: Lichinaceae)
Thallus fruticose, dry black, lobes erect surface smooth, cylindrical, central hyphal strand compact, photobiont a cyanobacterium. Apothecia terminal, emerging long, 0.5–1 mm in diameter, urceolate, disc concave to flat, black or dark brown, hymenium tainted, 100–120 µm high, IKI (+) blue, asci cylindrical clavate, 8 spored, from 50–70×15–22 µm, spores spherical or ellipsoidal, hyaline, 8–15×6–10 µm.

**Table 2:** Morpho-anatomical measurements and colorimetric characteristics of identified lichens.

Lichen species	Thallus size (cm)	Lobe width (mm)	Thallus upper surface color	Apothecia/Perithecia (mm)	Ascospore size (µm)
*Dermatocarpon vellereum*	5.5	55	Light brown	0.3	10.5×7.5
*Umbilicaria vellea*	3.5	35	Grey	-	-
*Rhizoplaca chrysoleuca*	3	30	Yellowish green	4	10.2×4.7
*Rhizoplaca melanophthalma*	2.7	27	Yellowish to brownish green	2.5	10.1×5.2
*Pleopsidium flavum*	4.5	0.8	Bright yellow	1	4.2×1.8
*Xanthoparmelia mexicana*	3.9	3	Light yellow green	-	-
*Acarospora badiofusca*	4.5	1	Reddish brown	0.9	4.5×2
*Xanthoria elegans*	6	0.9	Orange-red	1	14.5×8.2
*Lecanora frustulosa*	4.8	0.5	Greenish yellow	1.2	12.5×5.8
*Lobothallia alphoplaca*	8.2	1.2	Ashy grey	1.8	10.5×7.2
*Physconia muscigena*	7.8	1.2	Brownish white	3.6	27.5×14.1
*Melanelia disjuncta*	2.9	1.5	Dark brown	1.8	10.5×5.9
*Xanthoparmelia stenophylla*	10.6	3.5	Yellowish green	4.6	8.8×5.9
*Peccania coralloides*	2.5	1.4	Black	0.7	11.5×8.5

### Antioxidant capacities

#### Ferric reducing antioxidant power (FRAP)

The capacity of the lichen extracts to reduce ferric ions was evaluated by performing FRAP assay. In the present study, the extraction of antioxidants was done sequentially with n-hexane, methanol and water. All the lichen extracts exhibited high FRAP antioxidant capacity (Table 3). However, the rate of FRAP activity was found to vary with the lichen species and the extracting solvent. It was observed that the water extracts retained moderate FRAP values, while methanol extracts retained maximum FRAP and the n-hexane extracts retained least amount of FRAP for all lichen species. The methanol extracts of all lichens were found to possess significantly higher (*p*<0.05) FRAP values in comparison with the water and n-hexane extracts. Among all extracts, methanol extract of *L. alphoplaca* exhibited highest FRAP value (1673.32±94.83 µM Fe (II)/g extract). The maximum FRAP values in n-hexane and water extracts were observed in *X. elegans* (136.55±12.91 µM Fe (II)/g extract) and *P. coralloides* (335.19±12.92 µM Fe (II)/g extract), respectively. The positive standards *viz.*, ascorbic acid (9851.60±752.15 µM Fe (II)/g extract) and BHT (3160.14±251.72 µM Fe (II)/g extract) were found to exhibit significantly higher FRAP values than the lichen extracts (Table 3).

**Table 3:** Ferric reducing antioxidant power (FRAP) of high altitude cold desert saxicolous lichens^a^.

Lichen species	µM Fe (II)/g extract		
	Water	Methanol	n-Hexane
*Dermatocarpon vellereum*	178.15±8.83	1222.30±82.24*	89.45±7.81*^#^
*Umbilicaria vellea*	42.27±2.87	603.81±62.14*	61.43±5.88*^#^
*Rhizoplaca chrysoleuca*	95.57±6.02	1112.16±74.21*	108.87±10.56^#^
*Rhizoplaca melanophthalma*	133.31±8.62	648.08±67.82*	33.66±4.81*^#^
*Pleopsidium flavum*	70.58±4.86	618.56±59.98*	96.93±9.14*^#^
*Xanthoparmelia mexicana*	77.49±5.23	1479.63±85.12*	115.23±10.23*^#^
*Acarospora badiofusca*	123.02±8.26	1394.79±92.61*	114.16±12.66^#^
*Xanthoria elegans*	105.34±7.91	191.86±18.01*	136.55±12.91*^#^
*Lecanora frustulosa*	76.32±4.63	338.68±34.81*	74.02±6.86^#^
*Lobothallia alphoplaca*	47.25±2.39	1673.32±94.83*	52.40±6.11*^#^
*Physconia muscigena*	72.85±5.04	740.90±67.86*	127.64±10.86*^#^
*Melanelia disjuncta*	61.76±4.49	395.33±44.21*	98.12±8.68*^#^
*Xanthoparmelia stenophylla*	133.65±7.31	646.72±67.11*	37.38±4.45*^#^
*Peccania coralloides*	335.19±12.92	1353.46±84.18*	109.82±11.66*^#^
Ascorbic acid	9851.60±752.15		
BHT^b^	3160.14±251.72		

a Mean ±SD of three replicates;

b Butylated hydroxytoluene.

*p*<0.05: *compared with water extract; ^#^compared with methanol extract.

#### ABTS radical scavenging capacity

**Figure 2:**
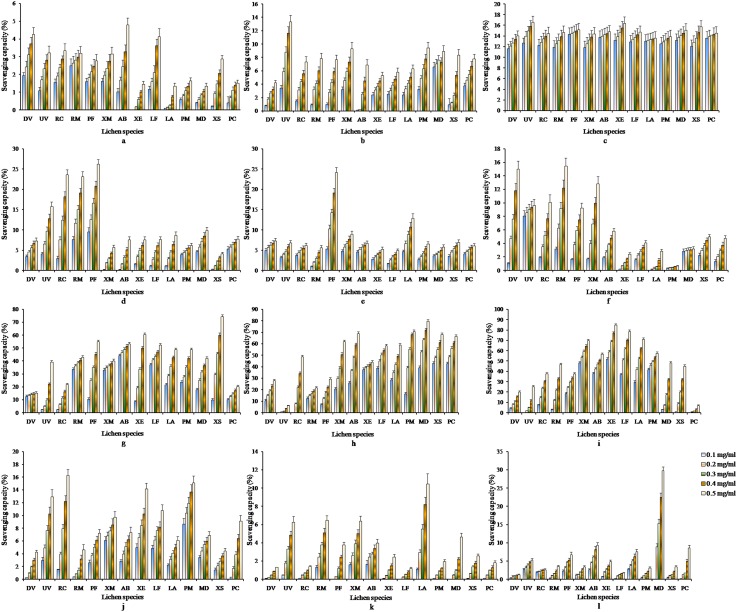
Effect of lichen extracts on various radical-scavenging capacities. **a**: ABTS radical scavenging capacity of n-hexane extracts; **b**: ABTS radical scavenging capacity of methanol extracts; **c**: ABTS radical scavenging capacity of water extracts; **d**: DPPH radical scavenging capacity of n-hexane extracts; **e**: DPPH radical scavenging capacity of methanol extracts; **f**: DPPH radical scavenging capacity of water extracts; **g**: Scavenging effect of n-hexane extracts on β-carotene-linoleic acid bleaching assay; **h**: Scavenging effect of methanol extracts on β-carotene-linoleic acid bleaching assay; **i**: Scavenging effect of water extracts on β-carotene-linoleic acid bleaching assay; **j**: Nitric oxide radical scavenging capacity of n-hexane extracts; **k**: Nitric oxide radical scavenging capacity of methanol extracts; **l**: Nitric oxide radical scavenging capacity of water extracts. **DV**: *Dermatocarpon vellereum*; **UV**: *Umbilicaria vellea*; **RC**: *Rhizoplaca chrysoleuca*; **RM**: *Rhizoplaca melanophthalma*; **PF**: *Pleopsidium flavum*; **XM**: *Xanthoparmelia mexicana*; **AB**: *Acarospora badiofusca*; **XE**: *Xanthoria elegans*; **LF**: *Lecanora frustulosa*; **LA**: *Lobothallia alphoplaca*; **PM**: *Physconia muscigena*; **MD**: *Melanelia disjuncta*; **XS**: *Xanthoparmelia stenophylla*; **PC**: *Peccania coralloides*.

The ABTS radical scavenging capacity (%) of the fourteen lichen species in different solvent extracts has been depicted in Fig. 2. The extracts scavenged the ABTS radical in a dose dependent manner at concentration of 0.1–0.5 mg/ml. The positive controls *viz*. quercetin and ascorbic acid at concentration of 0.1–0.5 mg/ml were also found to produce dose dependent inhibition of ABTS radical. The IC_50_ or RSa_50_ values of different solvent extracts and positive controls *viz.* quercetin and ascorbic acid were calculated in the present study and have been depicted in Table 4. The methanol extract of *X. stenophylla* showed the highest ABTS radical scavenging capacity (IC_50_ 1.88±0.09 mg/ml). The maximum ABTS radical scavenging capacity in n-hexane and water extracts was rendered by *A. badiofusca* (IC_50_ 6.55±0.10 mg/ml) and *U. vellea* (IC_50_ 3.45±0.11 mg/ml). ABTS radical scavenging capacity of methanol extracts was significantly higher (*p*<0.05) in comparison with water and n-hexane extracts for all the lichen species. Although *X. elegans* water extract (IC_50_ 5.08±0.10 mg/ml) showed significantly higher (*p*<0.05) radical scavenging capacity than the methanol and n-hexane extracts. The n-hexane extract of most of the lichen species under investigation exhibited significantly lower (*p*<0.05) antioxidant capacity when compared with the methanol and water extracts. However, the n-hexane extracts of *A. badiofusca* (IC_50_ 6.55±0.10 mg/ml), *L. alphoplaca* (IC_50_ 9.52±0.13 mg/ml) and *P. coralloides* (IC_50_ 14.84±0.09 mg/ml) showed significantly higher (*p*<0.05) antioxidant capacity in comparison with the corresponding water extracts. Quercetin (IC_50_ 0.33±0.03 mg/ml) and ascorbic acid (IC_50_ 0.18±0.01 mg/ml) were found to produce significantly higher (*p*<0.05) radical scavenging potential compared to the lichen extracts (Table 4).

**Table 4:** Scavenging effect of high altitude cold desert saxicolous lichens on ABTS and DPPH radicals^a^.

Lichen species	ABTS (IC_50_/RSa_50_), mg/ml			DPPH (IC_50_/RSa_50_), mg/ml		
	Water	Methanol	n-Hexane	Water	Methanol	n-Hexane
*Dermatocarpon vellereum*	6.39±0.10	6.24±0.10	8.12±0.10*^#^	1.59±0.11	7.85±0.10*	1.24±0.10*^#^
*Umbilicaria vellea*	3.45±0.11	1.92±0.11*	9.31±0.09*^#^	10.33±0.09	5.25±0.10*	1.23±0.10*^#^
*Rhizoplaca chrysoleuca*	7.61±0.10	3.45±0.09*	10.67±0.15*^#^	2.44±0.11	8.48±0.10*	1.02±0.02*^#^
*Rhizoplaca melanophthalma*	5.34±0.11	2.99±0.10*	26.50±0.10*^#^	1.65±0.09	3.91±0.02*	1.74±0.09^#^
*Pleopsidium flavum*	8.74±0.10	3.08±0.11*	16.47±0.09*^#^	2.54±0.10	1.09±0.01*	5.09±0.02*^#^
*Xanthoparmelia mexicana*	6.75±0.09	3.12±0.10*	12.52±0.10*^#^	1.97±0.08	4.58±0.11*	4.15±0.10*^#^
*Acarospora badiofusca*	14.60±0.10	2.45±0.09*	6.55±0.10*^#^	5.07±0.04	8.21±0.10^j^*	2.48±0.10*^#^
*Xanthoria elegans*	5.08±0.10	7.09±0.10*	11.78±0.10*^#^	7.71±0.11	8.10±0.10*	3.37±0.10*^#^
*Lecanora frustulosa*	8.72±0.10	5.71±0.11*	10.49±0.10*^#^	7.90±0.09	6.61±0.10*	3.04±0.03*^#^
*Lobothallia alphoplaca*	26.47±0.10	5.02±0.10*	9.52±0.13*^#^	4.13±0.11	2.29±0.01*	2.87±0.09*^#^
*Physconia muscigena*	11.10±0.11	3.15±0.08*	19.11±0.10*^#^	62.18±0.12	5.59±0.10*	8.62±0.10*^#^
*Melanelia disjuncta*	8.45±0.08	7.58±0.08*	21.67±0.10*^#^	67.44±0.10	8.40±0.10*	3.65±0.10*^#^
*Xanthoparmelia stenophylla*	4.42±0.10	1.88±0.09*	7.99±0.09*^#^	7.11±0.09	5.38±0.11*	5.08±0.02*^#^
*Peccania coralloides*	15.91±0.10	4.87±0.10*	14.28±0.09*^#^	5.95±0.05	10.25±0.09*	8.53±0.12*^#^
Quercetin	0.33±0.03^$^			0.08±0.02^$^		
Ascorbic acid	0.18±0.01^$^			0.05±0.01^$^		

a Mean ±SD of three replicates

*p*<0.05: *compared with water extract; ^#^compared with methanol extract; ^$^compared with all lichen extracts.

#### DPPH radical scavenging capacity

The free radical scavenging capacity of the fourteen lichen species in different extracts and the two positive controls *viz.* quercetin and ascorbic acid were compared through their ability to scavenge DPPH radical. The DPPH radical scavenging capacity of the lichen extracts and the positive controls increased in a dose dependent manner at concentration of 0.1–0.5 mg/ml. The half maximal inhibitory/scavenging concentration (IC_50_ or RSa_50_) values were determined and have been depicted in Table 4. Lower value of IC_50_ indicates higher DPPH radical scavenging capacity. The rate of DPPH radical scavenging capacity was found to depend on lichen species and extracting solvent. For most of the lichen species, DPPH radical scavenging capacity of n-hexane extract was significantly higher (*p*<0.05) in comparison with methanol and water extract. The radical scavenging capacity of n-hexane extracts of *D. vellereum* (IC_50_ 1.24±0.10 mg/ml), *U. vellea* (IC_50_ 1.23±0.10 mg/ml), *R. chrysoleuca* (IC_50_ 1.02±0.02 mg/ml), *A. badiofusca* (IC_50_ 2.48±0.10 mg/ml), *X. elegans* (IC_50_ 3.37±0.10 mg/ml), *L. frustulosa* (IC_50_ 3.04±0.03 mg/ml), *M. disjuncta* (IC_50_ 3.65±0.10 mg/ml) and *X. stenophylla* (IC_50_ 5.08±0.02 mg/ml) increased significantly (*p*<0.05) when compared with the methanol and water extracts. However, the water extract of *R. melanophthalma* (IC_50_ 1.65±0.09 mg/ml), *X. mexicana* (IC_50_ 1.97±0.08 mg/ml) and *P. coralloides* (IC_50_ 5.95±0.05 mg/ml) showed significant increase (*p*<0.05) in radical scavenging ability than n-hexane and methanol extract. In addition, the methanol extract of *L. alphoplaca* (IC_50_ 2.29±0.01 mg/ml), *P. muscigena* (IC_50_ 5.59±0.10 mg/ml) and *P. flavum* (IC_50_ 1.09±0.01 mg/ml) were found to produce significantly higher (*p*<0.05) radical scavenging capacity when compared with the corresponding n-hexane and water extracts. The n-hexane extract of *R. chrysoleuca* exhibited the highest radical scavenging capacity (IC_50_ 1.02±0.02 mg/ml) among all lichen extracts under our study. The maximum DPPH radical scavenging capacity in methanol and water extracts was retained by *P. flavum* (IC_50_ 1.09±0.01 mg/ml) and *D. vellereum* (IC_50_ 1.59±0.11 mg/ml), respectively. Quercetin (IC_50_ 0.08±0.02 mg/ml) and ascorbic acid (IC_50_ 0.05±0.01 mg/ml) were found to produce significantly higher (*p*<0.05) DPPH radical scavenging effect compared to the lichen extracts (Table 4).

#### β-carotene-linoleic acid bleaching assay

The β-carotene-linoleic acid bleaching capacity of different solvent extracts of fourteen lichen species was expressed as scavenging capacity (%). All the lichen extracts and positive control BHT showed β-carotene-linoleic bleaching activity in a dose dependent manner at concentration of 0.1-0.5 mg/ml and it was found to vary with extracting solvent and lichen species (Fig. 2). The IC_50_ or RSa_50_ values were determined for each lichen extract and BHT (Table 5). In the present study, the highest antioxidant capacity was observed in water extract of *X. elegans* (IC_50_ 0.08±0.01 mg/ml). The highest antioxidant capacity for n-hexane and methanol extracts was determined in *X. stenophylla* (IC_50_ 0.33±0.11 mg/ml) and *M. disjuncta* (IC_50_ 0.18±0.08 mg/ml) respectively. The methanol and water extracts of majority of the lichen species showed significantly higher (*p*<0.05) antioxidant capacity in comparison with the n-hexane extract. The methanol extracts of *D. vellereum* (IC_50_ 1.01±0.11 mg/ml), *R. chrysoleuca* (IC_50_ 0.52±0.11 mg/ml), *P. muscigena* (IC_50_ 0.22±0.08 mg/ml), *M. disjuncta* (IC_50_ 0.18±0.08 mg/ml), *P. coralloides* (IC_50_ 0.22±0.11 mg/ml), *X. stenophylla* (IC_50_ 0.22±0.10 mg/ml) and *A. badiofusca* (IC_50_ 0.32±0.10 mg/ml) were found to produce significantly higher (*p*<0.05) antioxidant capacity when compared with the corresponding n-hexane and water extracts. In addition, the water extracts of *X. mexicana* (IC_50_ 0.14±0.08 mg/ml), *L. frustulosa* (IC_50_ 0.20±0.10 mg/ml), *L. alphoplaca* (IC_50_ 0.28±0.09 mg/ml), *U. vellea* (IC_50_ 0.50±0.10 mg/ml), *R. melanophthalma* (IC_50_ 0.53±0.10 mg/ml) and *X. elegans* (IC_50_ 0.08±0.01 mg/ml) showed significantly higher (*p*<0.05) antioxidant capacity in comparison with the corresponding methanol and n-hexane extracts. However, *P. flavum* n-hexane extract (IC_50_ 0.45±0.10 mg/ml) showed significantly higher (*p*<0.05) antioxidant capacity than the corresponding methanol and water extracts. BHT was used as positive control and it showed significantly higher (*p*<0.05) antioxidant capacity (IC_50_ 0.04±0.01 mg/ml) in comparison with all lichen extracts (Table 5).

**Table 5:** Scavenging effect of high altitude cold desert saxicolous lichens on β-carotene-linoleic acid bleaching assay and nitric oxide radicals^a^.

Lichen species	β-carotene-linoleic acid bleaching assay (IC_50_/RSa_50_), mg/ml			Nitric oxide (IC_50_/RSa_50_), mg/ml		
	Water	Methanol	n-Hexane	Water	Methanol	n-Hexane
*Dermatocarpon vellereum*	1.33±0.10	1.01±0.11*	5.74±0.10*^#^	18.56±0.10	14.06±0.10*	6.29±0.10*^#^
*Umbilicaria vellea*	0.50±0.10	2.41±0.11*	0.57±0.08^#^	8.11±0.11	3.55±0.09*	2.11±0.11*^#^
*Rhizoplaca chrysoleuca*	0.56±0.09	0.52±0.11	1.14±0.10*^#^	28.33±0.09	17.91±0.11*	1.39±0.11*^#^
*Rhizoplaca melanophthalma*	0.53±0.10	1.85±0.09*	0.76±0.10*^#^	4.15±0.09	4.06±0.11	3.31±0.10*^#^
*Pleopsidium flavum*	0.76±0.09	0.89±0.10	0.45±0.10*^#^	3.98±0.09	4.37±0.07*	4.12±0.11^#^
*Xanthoparmelia mexicana*	0.14±0.08	0.38±0.05*	1.12±0.10*^#^	7.25±0.08	4.14±0.09*	5.09±0.10*^#^
*Acarospora badiofusca*	0.37±0.08	0.32±0.10	0.34±0.11	2.82±0.10	8.71±0.10*	4.27±0.07*^#^
*Xanthoria elegans*	0.08±0.01	0.88±0.10*	0.42±0.10*^#^	4.97±0.10	6.24±0.10*	2.75±0.08*^#^
*Lecanora frustulosa*	0.20±0.10	0.31±0.11	0.49±0.10*	7.21±0.11	18.61±0.10*	3.33±0.09*^#^
*Lobothallia alphoplaca*	0.28±0.09	0.39±0.10	0.50±0.10*	4.27±0.09	1.75±0.09*	5.24±0.11*^#^
*Physconia muscigena*	0.32±0.10	0.27±0.08	0.52±0.10^#^	4.62±0.10	13.59±0.10*	2.61±0.09*^#^
*Melanelia disjuncta*	0.54±0.08	0.18±0.08*	0.62±0.10^#^	0.75±0.09	11.20±0.10*	5.96±0.09*^#^
*Xanthoparmelia stenophylla*	0.62±0.11	0.22±0.11*	0.33±0.11*	3.26±0.07	8.69±0.11*	7.13±0.11*^#^
*Peccania coralloides*	1.71±0.10	0.22±0.10*	1.64±0.11^#^	1.46±0.13	12.56±0.10*	2.26±0.10*^#^
BHT^b^	0.04±0.01^$^			0.15±0.04^$^		

a Mean ±SD of three replicates; ^b^Butylated hydroxytoluene

*p*<0.05: *compared with water extract; ^#^compared with methanol extract; ^$^compared with all lichen extracts.

#### Nitric oxide scavenging assay

The scavenging capacity (%) of the different solvent extracts of fourteen lichen species against nitric oxide released by sodium nitroprusside was studied and the result has been depicted in Fig. 2. The percentage radical scavenging capacity of the extracts and the reference standard BHT against nitric oxide radical was increased in a dose dependent mode at concentration of 0.1–0.5 mg/ml. The IC_50_ or RSa_50_ values of lichen extracts and BHT were calculated and have been depicted in Table 5. *M. disjuncta* (IC_50_ 0.75±0.09 mg/ml) water extract showed the highest nitric oxide radical scavenging capacity among all lichen extracts under investigation. For n-hexane, methanol and water extracts, maximum radical scavenging capacity was detected in *R. chrysoleuca* (IC_50_ 1.39±0.11 mg/ml), *L. alphoplaca* (IC_50_ 1.75±0.09 mg/ml) and *M. disjuncta* (IC_50_ 0.75±0.09 mg/ml) respectively. Majority of the lichen species were found to produce significantly higher (*p*<0.05) nitric oxide radical scavenging capacity in their n-hexane and and water extracts in comparison with corresponding methanol extracts. The n-hexane extracts of *D. vellereum* (IC_50_ 6.29±0.10 mg/ml), *U. vellea* (IC_50_ 2.11±0.11 mg/ml), *R. chrysoleuca* (IC_50_ 1.39±0.11 mg/ml), *R. melanophthalma* (IC_50_ 3.31±0.10 mg/ml), *X. elegans* (IC_50_ 2.75±0.08 mg/ml), *L. frustulosa* (IC_50_ 3.33±0.09 mg/ml) and *P. muscigena* (IC_50_ 2.61±0.09 mg/ml) were found to exhibit significantly higher (*p*<0.05) radical scavenging capacity in comparison with the corresponding water and methanol extracts. In addition, the water extracts of *P. coralloides* (IC_50_ 1.46±0.13 mg/ml), *X. stenophylla* (IC_50_ 3.26±0.07 mg/ml), *M. disjuncta* (IC_50_ 0.75±0.09 mg/ml), *A. badiofusca* (IC_50_ 2.82±0.10 mg/ml) and *P. flavum* (IC_50_ 1.01±0.11 mg/ml) showed significantly increased (*p*<0.05) radical scavenging capacity in comparison with the corresponding n-hexane and methanol extracts. However, the methanol extracts of *X. mexicana* (IC_50_ 4.14±0.09 mg/ml) and *L. alphoplaca* (IC_50_ 1.75±0.09 mg/ml) were found to produce significantly higher (*p*<0.05) radical scavenging capacity in comparison with the corresponding n-hexane and water extracts. BHT showed significantly higher (*p*<0.05) nitric oxide radical scavenging capacity (IC_50_ 0.15±0.04 mg/ml) in comparison with all lichen extracts (Table 5).

### Phytochemical compositions

#### Total proanthocyanidin content (TPAC)

The TPAC of the different lichen extracts was expressed as mg CAE/100 g dry weight (dw) of extract. The methanol extracts of all lichen species were found to contain significantly higher (*p*<0.05) TPAC in comparison with the corresponding n-hexane and water extracts. Methanol extract of *X. mexicana* showed highest TPAC (4218.59±5.21 mg CAE/100 g) among all lichen extracts. The maximum TPAC in the n-hexane and water extracts was found in *M. disjuncta* (178.52±1.56 mg CAE/100 g) and *P. coralloides* (1071.82±0.64 mg CAE/100 g) respectively (Table 6).

**Table 6:** Total proanthocyanidins (TPAC), flavanoids (TFC), and phenolic contents (TPC) in high altitude cold desert saxicolous lichens^a^.

Lichen species	TPAC (mg CAE/100 g dw)			TFC (mg QAE/100 g dw)			TPC (mg GAE/100 g dw)		
	Water	Methanol	n-Hexane	Water	Methanol	n-Hexane	Water	Methanol	n-Hexane
*Dermatocarpon vellereum*	181.04±23.03	1506.34±142.65*	120.24±14.93*^#^	104.82±9.44	296.11±26.35*	77.00±6.82*^#^	471.19±20.46	2423.12±71.61*	158.32±7.18*^#^
*Umbilicaria vellea*	191.24±30.22	1356.98±132.75*	143.41±12.72*^#^	43.15±6.26	812.07±77.60*	80.20±9.89*^#^	240.80±14.14	2812.92±78.56*	151.69±8.87*^#^
*Rhizoplaca chrysoleuca*	152.82±18.62	709.93±78.83*	144.11±15.93*^#^	74.74±8.59	532.01±61.51*	138.03±12.43*^#^	228.07±12.62	2786.48±79.82*	255.7±10.58*^#^
*Rhizoplaca melanophthalma*	138.87±15.68	1202.16±148.54*	70.25±8.53*^#^	84.54±9.70	647.38±68.48*	62.68±8.54*^#^	272.30±11.68	2148.85±66.12*	96.37±6.55*^#^
*Pleopsidium flavum*	146.88±16.62	521.57±46.25*	135.49±16.09*^#^	66.45±7.52	272.54±26.97*	162.90±31.58*^#^	184.35±12.50	2096.01±68.20*	174.78±9.27*^#^
*Xanthoparmelia Mexicana*	195.24±25.51	4218.59±454.21*	151.01±16.93*^#^	142.68±15.50	3607.56±319.18*	154.70±14.71*^#^	169.87±10.52	5439.93±61.45*	226.31±11.40*^#^
*Acarospora badiofusca*	184.25±24.67	1303.54±152.62*	127.68±13.52*^#^	113.02±12.83	663.63±71.33*	122.74±10.40*^#^	324.95±14.73	2816.60±88.59*	171.91±10.56*^#^
*Xanthoria elegans*	176.01±22.01	1228.56±124.09*	162.53±14.38*^#^	117.06±11.01	256.85±20.21*	158.25±23.28*^#^	241.78±12.08	1974.04±51.98*	203.42±10.11*^#^
*Lecanora frustulosa*	156.27±14.59	638.56±66.78*	144.98±15.03*^#^	63.28±8.70	357.52±30.73*	202.19±30.97*^#^	192.56±8.56	1422.16±46.76*	212.31±11.43*^#^
*Lobothallia alphoplaca*	121.64±15.28	1409.38±144.93*	69.09±9.74*^#^	33.49±4.18	522.38±61.43*	64.18±5.91*^#^	157.89±8.17	2284.40±63.81*	81.62±6.82*^#^
*Physconia muscigena*	135.25±12.60	1241.10±138.07*	154.03±18.97*^#^	58.32±7.57	363.15±40.23*	150.28±15.12*^#^	196.27±9.58	2496.12±69.56*	196.36±12.86*^#^
*Melanelia disjuncta*	167.47±20.40	844.53±83.43*	178.52±24.56*^#^	88.15±9.44	528.29±48.35*	140.64±16.44*^#^	261.22±11.39	2522.67±64.54*	198.03±12.51*^#^
*Xanthoparmelia stenophylla*	302.18±36.65	1521.79±158.46*	64.85±8.57*^#^	97.81±10.60	1456.46±131.17*	95.98±11.57*^#^	332.46±19.49	1891.28±38.04*	162.78±10.60*^#^
*Peccania coralloides*	509.82±64.64	1648.33±162.32*	143.18±15.67*^#^	122.97±14.54	1444.84±152.48*	142.03±12.22*^#^	604.07±21.67	2745.55±81.41*	180.28±12.86*^#^

a Mean ±SD of three replicates

*p*<0.05: *compared with water extract; ^#^compared with methanol extract.

#### Total flavonoid content (TFC)

The TFC was expressed as mg QAE/100 g dw of extract. In our present investigation, the methanol extracts of all lichen species were found to have significantly higher (*p*<0.05) TFC in comparison with the corresponding n-hexane and water extracts. The methanol extract of *X. mexicana* showed highest TFC (3607.56±4.18 mg QAE/100 g) among all lichen extracts. The maximum TFC in n-hexane and water extracts was determined in *L. frustulosa* (202.19±30.97 mg QAE/100 g) and *X. mexicana* (142.68±15.50, mg QAE/100 g) respectively (Table 6).

#### Total polyphenol content (TPC)

The TPC of the lichen extracts was expressed as mg GAE/100 g dw of extract. In our current investigation, the methanol extracts of all lichen species were found to have significantly higher (*p*<0.05) TPC in comparison with the corresponding n-hexane and water extracts. The methanol extract of *X. mexicana* showed highest TPC (5439.93±61.45 mg GAE/100 g) among all lichen extracts. The maximum TPC in n-hexane and water extracts was determined in *R. chrysoleuca* (255.70±10.58 mg GAE/100 g) and *P. coralloides* (604.07±21.67 mg GAE/100 g) respectively (Table 6).

### Cytotoxic effects of lichen extracts

Depending on high antioxidant capacities, we have screened eight lichen extracts [LW1: water extract of *D. vellereum*, LW2: water extract of *U. vellea*, LW8: water extract of *X. elegans*, LW12: water extract of *M. disjuncta*, LM5: methanol extract of *M. disjuncta*, L10M: methanol extract of *L. alphoplaca*, L12M: methanol extract of *M. disjuncta*, L13M: methanol extract of *X. stenophylla*] out of the total fourty two lichen extracts to investigate the cytotoxicity and antiproliferative activities in cellular model.

#### HepG2 cells

**Figure 3:**
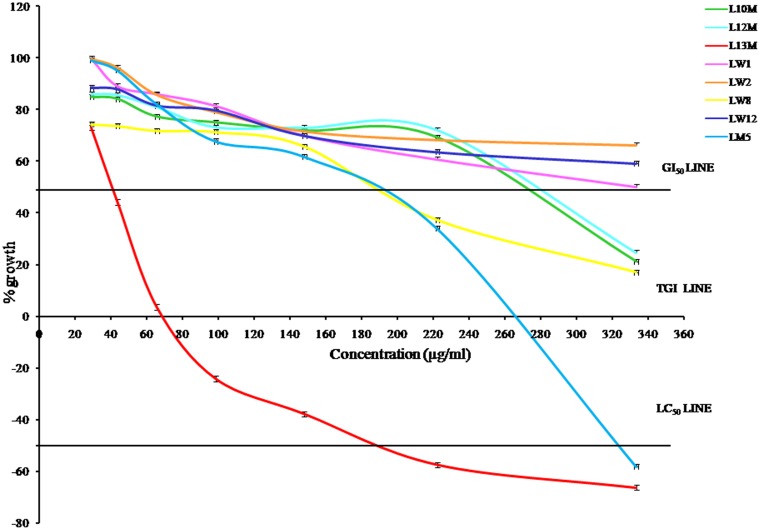
Cytotoxic effect of lichen extracts on HepG2 cells. LW1: water extract of *D. vellereum*, LW2: water extract of *U. vellea*, LW8: water extract of *X. elegans*, LW12: water extract of *M. disjuncta*, LM5: methanol extract of *M. disjuncta*, L10M: methanol extract of *L. alphoplaca*, L12M: methanol extract of *M. disjuncta*, L13M: methanol extract of *X. stenophylla*.

In HepG2 cells, extract LW2 and LW12 showed no toxicity as they remained far above from GI_50_. The % growth with treatment of LW2 and LW12 at highest concentration level 333.33 µg/ml was 66.22±3.88 and 59.171±4.33 respectively (Fig. 3). LW1 was found to be less toxic as it approached the GI_50_ at 333.33 µg/ml (% growth 49.967±3.81) and all concentrations were non-toxic below this level. L10M and L12M crossed the GI_50_ at 275 µg/ml (% growth 21.28±1.94) and 281 µg/ml (% growth 24.61±1.87) and were found to be toxic. LW1, L10M and L12M were non-toxic upto 222.22 µg/ml. LW8 was very toxic at 333.33 µg/ml where it approached almost near to GI_90_ and crossed GI_50_ at 189 µg/ml. L13M and LM5 were extremely toxic for HepG2 cells as they crossed the GI_50_ at 41 µg/ml and 191 µg/ml, TGI at 69 µg/ml and 266 µg/ml, and LC_50_ at 190 µg/ml and 324 µg/ml respectively. They showed −66.31±4.52% and −58.19±4.54% growth at 333.33 µg/ml. All these results have been depicted in Fig. 3.

#### RKO cells

**Figure 4:**
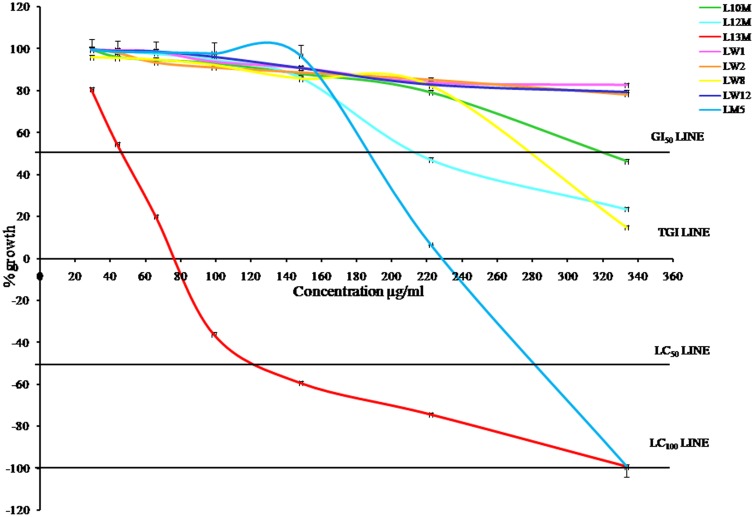
Cytotoxic effect of lichen extracts on RKO cells. LW1: water extract of *D. vellereum*, LW2: water extract of *U. vellea*, LW8: water extract of *X. elegans*, LW12: water extract of *M. disjuncta*, LM5: methanol extract of *M. disjuncta*, L10M: methanol extract of *L. alphoplaca*, L12M: methanol extract of *M. disjuncta*, L13M: methanol extract of *X. stenophylla*.

In case of RKO cells, LW1, LW2 and LW12 were non-toxic at all concentration levels. However, LW8 and L10M crossed GI_50_ at 289 and 330 µg/ml. The percent growth recorded with the treatment of LW8 and L10M extracts at highest concentration 333.33 µg/ml was 14.77±1.87 and 46.25±3.53 respectively. L12M was toxic as it crossed GI_50_ at 213 µg/ml, cells showed 23.58±2.15% growth at 333.33 µg/ml and it was non-toxic upto148.15 µg/ml concentration. LM5 and L13M were toxic as they approached the LC_99_ at 333.33 µg/ml and showed −99.21±0.88% and −99.28±0.38% growth respectively. LM5 crossed the LC_50_, TGI and GI_50_ at 282, 229 and 187 µg/ml respectively. L13M crossed the LC_50_, TGI and GI_50_ at 121, 76 and 46 µg/ml respectively. All these results have been depicted in Fig. 4.

#### Morphology of treated cells by microscopic analysis

In morphological visualization after 24, 48 and 72 h, extract LW1, LW2 and LW12 were non toxic at all concentration levels whereas L13M was highly toxic as it showed toxicity up to 98.77 µg/ml for HepG2 cell line and up to 65.84 µg/ml for RKO cell line. Extract LW8, L5M and L12M were non toxic up to 222.22, 148.15 and 148.45 µg/ml, respectively, for both cell lines. L10M was found to be non toxic up to 222.22 µg/ml for HepG2 cell line and up to 148.15 µg/ml for RKO cell line. In HepG2 cell line, extract LM5 showed dead cells at 333.33 µg/ml after 24, 48 and 72 h; and 50% dead cells at 222.22 µg/ml after 24 h. L12M showed 50% cell death at 333.33 µg/ml after 24 and 48 h. L13M showed dead cells at 222.22 and 333.33 µg/ml after 24 h, at 98.77 to 333.33 µg/ml after 48 h and at 148.15 to 333.33 µg/ml after 72 h. In RKO cells, L13M showed dead cells at 222.22 and 333.33 µg/ml after 24 h, at 98.77 to 333.33 µg/ml after 48 h and at 148.15 to 222.22 µg/ml after 72 h. LM5 showed dead cells at 333.33 µg/ml after 48 and 72 h. All results related to the morphological analysis have been depicted in Table 7.

**Table 7:** Cytotoxicity of lichen extract examined by microscopic visualization (10X eyepiece and 10X objective lenses) to investigate cell density and cell health.

HepG2 carcinoma cells																												
S. No	Conc. (µg/ml)	C			LW1			LW2			LW8			LW12			LM5			L10M			L12M			L13M		
		24 h	48 h	72 h	24 h	48 h	72 h	24 h	48 h	72 h	24 h	48 h	72 h	24 h	48 h	72 h	24 h	48 h	72 h	24 h	48 h	72 h	24 h	48 h	72 h	24 h	48 h	72 h
1	333.33	nt	nt	nt	nt	nt	nt	nt	nt	nt	gafp	nt	ga	nt	nt	nt	d	d	d	gafp	nt	ga	dfp	dfp	ga	d	d	d
2	222.22	nt	nt	nt	nt	nt	nt	nt	nt	nt	nt	nt	nt	nt	nt	nt	dfp	nt	nt	nt	nt	nt	ga	nt	nt	d	d	d
3	148.15	nt	nt	nt	nt	nt	nt	nt	nt	nt	nt	nt	nt	nt	nt	nt	nt	nt	nt	nt	nt	nt	nt	nt	nt	ga	d	d
4	98.77	nt	nt	nt	nt	nt	nt	nt	nt	nt	nt	nt	nt	nt	nt	nt	nt	nt	nt	nt	nt	nt	nt	nt	nt	ga	dfp	ga
5	65.84	nt	nt	nt	nt	nt	nt	nt	nt	nt	nt	nt	nt	nt	nt	nt	nt	nt	nt	nt	nt	nt	nt	nt	nt	nt	nt	nt
6	43.90	nt	nt	nt	nt	nt	nt	nt	nt	nt	nt	nt	nt	nt	nt	nt	nt	nt	nt	nt	nt	nt	nt	nt	nt	nt	nt	nt
7	29.26	nt	nt	nt	nt	nt	nt	nt	nt	nt	nt	nt	nt	nt	nt	nt	nt	nt	nt	nt	nt	nt	nt	nt	nt	nt	nt	nt

RKO carcinoma cells																												
S. No	Conc. (µg/ml)	C			LW1			LW2			LW8			LW12			LM5			L10M			L12M			L13M		
		24 h	48 h	72 h	24 h	48 h	72 h	24 h	48 h	72 h	24 h	48 h	72 h	24 h	48 h	72 h	24 h	48 h	72 h	24 h	48 h	72 h	24 h	48 h	72 h	24 h	48 h	72 h
1	333.33	nt	nt	nt	nt	nt	nt	nt	nt	nt	nt	nt	ga	nt	nt	nt	ga	dsp	d	ga	ga	ga	ga	ga	ga	d	d	d
2	222.22	nt	nt	nt	nt	nt	nt	nt	nt	nt	nt	nt	nt	nt	nt	nt	nt	nt	ga	gafp	gafp	nt	gafp	nt	nt	d	d	d
3	148.15	nt	nt	nt	nt	nt	nt	nt	nt	nt	nt	nt	nt	nt	nt	nt	nt	nt	nt	nt	nt	nt	nt	nt	nt	ga	d	d
4	98.77	nt	nt	nt	nt	nt	nt	nt	nt	nt	nt	nt	nt	nt	nt	nt	nt	nt	nt	nt	nt	nt	nt	nt	nt	gafp	dep	ga
5	65.84	nt	nt	nt	nt	nt	nt	nt	nt	nt	nt	nt	nt	nt	nt	nt	nt	nt	nt	nt	nt	nt	nt	nt	nt	nt	ga	nt
6	43.90	nt	nt	nt	nt	nt	nt	nt	nt	nt	nt	nt	nt	nt	nt	nt	nt	nt	nt	nt	nt	nt	nt	nt	nt	nt	nt	nt
7	29.26	nt	nt	nt	nt	nt	nt	nt	nt	nt	nt	nt	nt	nt	nt	nt	nt	nt	nt	nt	nt	nt	nt	nt	nt	nt	nt	nt

c: control, nt: non-toxic, ga: growth arrested, gafp: growth arrested 50%, dfp: death 50%, dsp: death 70%, dep: death 80%, d: dead.

## Discussion

Lichens, one of the prominent life-form in the trans-Himalayan Ladakh region contain natural antioxidants which provide protection from the damage caused by the climatic conditions of high altitude environments and support their adaptability in extreme environmental conditions like extreme habitats (rocks in high altitude cold desert), low temperature, drought, prolonged winter, high solar irradiance and ultraviolet radiations. Numerous extracellular deposits in lichen thallus may contribute to the activation of antioxidant defence system in these conditions. In addition, diverse secondary metabolites are synthesized by lichens that are unique and found rarely in other plants [63]. In recent time, much attention has been paid on the biological roles of lichen secondary substances that have been found to have a lot of positive biological activities *viz.* antitumour, antibacterial, antifungal, antiviral, antiinflammatory and antioxidant capacities [64]. Therefore the secondary metabolites of high altitude cold desert lichens might be a potential resource of natural antioxidants and deserved to be tested for their antioxidant capacities.

The antioxidant capacities of plant extracts have been ascribed to various mechanisms of actions such as deterrence of chain initiation, radical scavenging activity, binding of transition metal ion catalysts, increasing endogenous status of antioxidant enzymes etc to prevent oxidative damage [65]. In order to understand the different mechanism of action of antioxidants, several antioxidant assays have been developed because the use of more than one assay would give a better insight into the true antioxidant potential of the test samples. Hence, it is important to use different assays for evaluating the antioxidant capacities of plant extracts that may have different mechanisms of antioxidant actions [66]. Various mechanisms of antioxidant action along with a range of methods of initiation, detection and measurement of oxidative processes *in vitro* and *in vivo* suggest probable explanations for structure-activity relationships. In general, the antioxidant capacity is known to be associated with the species, extraction solvent, method of extraction, temperature, conditions of the test systems, composition of the extract and hydrophobic or hydrophilic nature of the antioxidants. In our result, a wide range of antioxidant properties in different lichen extracts were observed that may be attributed to presence of diverse antioxidant compounds with different polarity in these lichen species. The bioactive antioxidants in the lichen extracts showed their diverse antioxidative effects in different antioxidant assays depending upon their individual redox reactions that may differ from each other by different magnitude of electrostatic interactions. In agreement with previous findings, the present study also revealed that the lichen extracts are the complex mixture of different compounds with distinct antioxidant capacities [67], [68]. In our result, different trends of antioxidant and radical scavenging capacities of different lichen extracts were observed which may be due to the presence of different types of compounds in these lichen species. The resulting antioxidant action depends on the individual redox reactions of bioactive compounds present in the lichens and for this reason it may differ from each other by different magnitude of molecular interactions. The antioxidant capacities of the studied lichen extracts were found to be dependent on species type and polarity of extracting solvent.

Previous reports on lichen diversity in the Antarctic regions revealed that lichens are well adapted for extreme climatic conditions and they can tolerate long periods of drought, severe cold and arid atmosphere. They have developed protective mechanisms to evade damage caused by high radiation and freezing temperatures [69]–[71] and all these adaptations have positive influence for their growth and survival in response to climate change and anthropogenic activities. There is dearth of information about the lichen species of the trans-Himalayan cold desert and it is therefore necessary to access the diversity, occurrence of potential bioactive compounds and antioxidant capacity of the trans-Himalayan lichens with considerable details. Lichens are the prominent life-form in the trans-Himalayan cold desert that produce diverse natural antioxidants having protective action against stressful environments of high altitude and also account for their adaptability in extreme climatic conditions. The numerous extracellular deposits in lichens may potentially contribute to the overall antioxidant protection [72]. The bioactive antioxidants produced by lichens also provide protection from ultra violet B (UV-B) radiation [70]. In Antarctica, the antioxidant activity in lichens is used as a measure of UV-B radiation that reaches the surface of the terrestrial environment. In the present study, we also found strong antioxidant capacity in the lichen extracts of trans-Himalayan high altitude that is in concordance with the previous reports on antioxidant content in Antarctic lichens [27], [70].

Researchers pay keen attention on the biological roles of lichen secondary substances that are reported to have biological activities such as antitumour, antibacterial, antifungal, antiviral, antiinflammatory and antioxidant activities [73], [74]. Interestingly, we observed higher antioxidant capacity in some lichen extracts in comparison with the antioxidant capacities of native plants of trans-Himalayan cold desert [48], [75], that signifies the importance of lichens as therapeutic agents. Therefore, the trans-Himalayan saxicolous lichens might be a potential resource of natural antioxidants for their high antioxidant capacities that may be attributed to the elevated content of diverse secondary metabolites.

Biologically active phytochemicals like polyphenols, flavonoids, flavonols, proanthocyanidins, alkaloids, terpenoids, carotenoids etc. are derived from plant sources and natural products which have promising health promoting properties and protective effects against chronic diseases while acting in combination [76]. Phenolics are the major secondary metabolites from plant sources that play crucial function in the regulation of plant growth and development in stressful and unfavourable climatic conditions. Lichens of the trans-Himalayan cold desert grow in extreme environmental conditions with severe cold, arid and water scarcity which in turn could cause up-regulated pathways of secondary metabolite synthesis and increased production of polyphenolics and other unique secondary metabolites that have protective effects against oxidative stress owing to their antioxidant capacities [34], [72], [77], [78].

It is well established that the antioxidant capacity of plant is associated with the phenolic compounds [79]. On the other hand, few studies revealed no correlation between antioxidant activity and TFC and TPC content of the extracts [80]–[83]. Therefore, it is important to investigate the effect of total phenolic content on the antioxidant capacity of the studied lichen extracts. Strong positive correlations were found among the antioxidant capacity assays (ABTS, DPPH, β-carotene-linoleic acid bleaching, nitric oxide, FRAP) and phenolic contents (TPAC, TFC, TPC) in all lichen extracts. Therefore, in agreement with previous reports, the results on correlations of different antioxidant assays and total phenolic content have demonstrated that the antioxidant capacity of the lichen extracts was positively associated with total phenolic content [33], [34], [78], [79], [84]. Thus, the present study highlights the antioxidant capacities rendered by the lichen extracts under oxidative stress.

In recent time a good number of research investigations were carried out to establish the positive biological properties of lichen extracts and their metabolites in different assays like antimicrobial [85]–[90], antioxidant [87]–[93], cytotoxic and antiproliferative [85], [86], [88], [94]–[100], antiviral [101]–[103], genotoxic and anticancer [91], [96], [97], antimycotic, antiinflammatory, analgesic, antipyretic, antiradiation, allelochemical, antiherbivore etc. [104]–[106]. Hence, lichens can be utilized as a natural bioresource for antioxidant and cytotoxic agent due to their promising biological activity and protective antistress function. At the same time, the *in vitro* antioxidant capacity assays obtained by the available chemical tests are not sufficient to prove the medicinal and therapeutic potential of these natural resources unless they are supported by the scientific data with the cytoprotective effects of these compounds in cell culture system. Therefore, the antioxidant capacity and cytotoxic effect of a given compound should be studied directly in a cellular model as it provides the optimal environment for possible interaction of the intracellular components with the compound of interest that would be missed in a chemical assay system. Moreover, we must be well aware of the safety of these lichen extracts because of the fact that many natural antioxidant may act as pro-oxidant based on the dose and on the ambient redox conditions [107], they may have different means for exerting their action, including interactions with intracellular signal transduction machineries and/or inducing the expression of antioxidant and detoxification enzymes [108] and could act as an anticancer agent [109]–[111]. In this milieu, we extend our present study towards the evaluation of cytotoxicity of eight lichen extracts out of the total fourty two extracts of fourteen lichen species those were taken for the initial screening based on antioxidant capacities and bioactive phytochemical constituents (the lichen extracts possessing high antioxidant capacities and potential phytocompounds were selected for cell line study) on human hepatocellular carcinoma HepG2 and colon carcinoma RKO cells.

**Figure 5:**
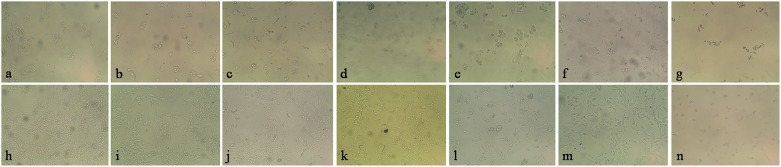
Microscopic images (10X eyepiece and 10X objective lenses) showing cell growth and morphology after 72 h treatment of lichen extracts in HepG2 and RKO carcinoma cells. a. HepG2 cells with 90% growth, **b.** HepG2 cells with 70% arrested growth, **c.** HepG2 cells with 80% arrested growth, **d.** HepG2 cells with 99% arrested growth, **e.** HepG2 cells under stress, **f.** HepG2 cells under stress near death, **g.** HepG2 dead cells, **h.** RKO cells with 90% growth, **i.** RKO cells with 50% arrested growth, **j.** RKO cells with 70% arrested growth, **k.** RKO cells with 80% arrested growth, **l.** RKO cells with 99% arrested growth, **m.** RKO cells under stress, **n.** RKO dead cells.

In comparison to RKO cells, HepG2 cells were more susceptible to LW1, LW2, LW8 and LW12 and the RKO cells were mostly susceptible to the remaining extracts (Table 7, Fig. 3–5). LW1 was toxic to HepG2 cells as it approached GI_50_ at 333.33 µg/ml, whereas it was non-toxic to RKO cells as there was 82.68±3.78% growth at the same concentration. The 222.22 µg/ml concentration of LW8 was toxic for HepG2 cells as there was 37.24±3.55% growth at this level whereas RKO cells was non-toxic at the same concentration level with 82.2±4.21% growth. The lichen *X. elegans* (LW8) are edible as medicine [37]–[43] and from this result, it was apparent that extract LW8 with 222.22 µg/ml concentration level could be used as anticancer agent for HepG2 cells along with gamma radiation in order to reduce the dose of radiation to avoid damage to normal cells. Moreover, extract LM5 and L13M can be used for further trial in search of potent sensitizer depending upon their LC_50_ values against both types of carcinoma cells, whereas LW1, LW2, LW8 and LW12 can be used for further trial in search of potent radioprotector at the safe and non-toxic concentration levels considered under the present study. Hence, from the results of cytotoxic effects of lichen extracts in carcinoma cells we observed that the methanol extract of *L. alphoplaca* and *M. disjuncta* were exhibiting high cytotoxic effects against the cancer cell growth and these extracts hold immense potential for use as anticancer agents. However identification of potential bioactive compound needs to be done in different extracts. Similarly, the water extract of *D. vellereum*, *U. vellea*, *X. elegans* and *M. disjuncta* and the methanol extract of *M. disjuncta* and *X. stenophylla* showed high antioxidant capacities and were found to be non-toxic and may be used as natural antioxidants for stress related problems.

The results of cytotoxicity assay revealed that the selected lichen extracts used in the present study exert cytotoxicity and antiproliferative action on HepG2 and RKO cell lines (Fig. 3–5, Table 7). The loss of viability of the dying cells as evidenced by the morphological changes was scrutinized by microscopy. Depending on the concentration, the lichen extracts exhibited different levels of cytotoxicity like cell shrinkage, aggregation and cell death (Fig. 5). In accordance with National Cancer Institute (NCI) guideline, the crude extract is considered active if the GI_50_ value <100 µg/ml [112], [113]. From our result in both cell lines, it was clear that GI_50_ of L13M was well below 100 µg/ml and that of LM5 was above 100 µg/ml, but at 333.33 µg/ml concentration both crude extracts showed almost equal percent of deaths of cells. All selected lichen extracts exhibited cytotoxic effect with increased cell death whereas reduction of cytotoxicity was observed with reduced extract concentration which finds support to the previous investigations [114].

Our results corroborate the previous investigations where cytotoxic and anticancer action of lichen extracts were explored in different lichen species from different parts of the world [91], [96], [98], [99], [106]. In agreement with our results, protective effects of *U. vellea* and *X. elegans* extracts against cellular damage by environmental stressors and survival potential of these lichens under extreme environmental condition was reported previously [100], [115]–[123]. However, the biological properties of other lichens remain to be investigated and to the best of our knowledge, this is the first study to explore the medicinal and therapeutic activities of trans-Himalayan lichen species.

## Conclusions

In the present study, fourteen saxicolous lichens from trans-Himalayan cold desert of Ladakh have been identified with their morpho-anatomical and chemical compositions and the antioxidant and free radical scavenging capacities of these lichen extratcts were reported for the first time. This study revealed that these lichen species have broad spectrum free radical scavenging effect and high antioxidant capacity. Moreover, the antioxidant capacity was found to be associated with the species and the polarity of solvent used for extraction. Our studies clearly showed that the high altitude saxicolous lichens can be an interesting source of new antioxidative substrates with the potential to be used for scavenging various types of free radicals by different scavenging mechanisms. The lichen extracts were found to contain considerable amount of phenolic compounds which were responsible for their high antioxidant and free radical scavenging ability and could be used as natural source of antioxidants to ameliorate oxidative stress related disorders. Among all extracts, eight lichen extracts having high antioxidant capacity and phenolic compounds were further investigated for cytotoxic action on human carcinoma cell lines and these extracts were found to exhibit anticancer and radioprotective effects that would be of great interest for further chemical investigations and could lead to the discovery of novel cytotoxic anticancer molecules from lichens of the trans-Himalayan region. With these present primary findings, further studies are also required to ascertain the biological activities of the lichen extracts in animal models.
